# Sustainable Biochar–Alumina Composites for Electroanalytical Sensing of Herbicide and Antibiotic

**DOI:** 10.3390/jox15060191

**Published:** 2025-11-10

**Authors:** Nataša Jović-Jovičić, Tatjana Novaković, Tanja Barudžija, Marija Ajduković, Natalia Czerwinska, Chiara Giosuè, Zorica Mojović

**Affiliations:** 1 University of Belgrade-Institute of Chemistry, Technology and Metallurgy, Department of Catalysis and Chemical Engineering, Njegoševa 12, 11000 Belgrade, Serbia; natasa.jovicjovicic@ihtm.bg.ac (N.J.-J.); tatjana.novakovic@ihtm.bg.ac.rs (T.N.); marija.ajdukovic@ihtm.bg.ac.rs (M.A.); 2 Vinča Institute of Nuclear Sciences, National Institute of RS, University of Belgrade, Mike Petrovića Alasa 12-14, 11000 Belgrade, Serbia; tbarudzija@vin.bg.ac.rs; 3 Università Politecnica delle Marche UnivPM, INSTM Research Unit-Department of Materials, Environmental Sciences and Urban Planning SIMAU, via Brecce Bianche 12, 60131 Ancona, Italy; n.czerwinska@staff.univpm.it (N.C.); c.giosue@univpm.it (C.G.)

**Keywords:** biochar–alumina composites, electrochemical sensor, pendimethalin, ciprofloxacin

## Abstract

The problem of water pollution by various xenobiotics has gained a lot of interest due to their persistence, bioaccumulation potential, and toxic effects on ecosystems and humans. Electrochemical sensors offer a rapid, sensitive, and cost-effective method for on-site monitoring. In this research, an electrochemical sensor for xenobiotics based on a biochar–alumina composite is developed. The biochar–alumina composites were obtained by the air-limited pyrolysis of oak sawdust in the presence of alumina. Two types of alumina were mixed with oak sawdust in three ratios and subjected to thermal treatment. The resulting composites were characterized by SEM, N_2_ adsorption isotherm, XRD, and electrochemical characterization. The detection of the herbicide pendimethalin and the antibiotic ciprofloxacin was investigated, and the composite with the optimal biochar/alumina ratio was selected for each of the xenobiotics studied. A linear current response was obtained for pendimethalin in the concentration range 0.7 μM to 70.0 μM with an LOD of 0.5 μM. A linear current response was obtained for ciprofloxacin in the concentration range 1.6 μM to 55.4 μM with an LOD of 0.63 μM. A comparison of the characterization results with the electroanalytical performance implied the importance of the hydrophobic/hydrophilic nature of the electrode surface for detecting the analyte under investigation.

## 1. Introduction

The pollution of water with various organic compounds is one of the prominent environmental problems in modern society. These organic compounds have multiple origins. Among anthropogenic chemicals, pesticides and antibiotics rank among the most extensively produced and applied globally, leading to widespread environmental dissemination. The application of pesticides in agriculture and antibiotics in health care has well-known benefits. However, even with caution and the proper use of these compounds, their presence in various waters is almost unavoidable.

Due to their use in agriculture and medicine, the herbicide pendimethalin and the antibiotic ciprofloxacin are significant xenobiotics that are commonly found in soil and water. Pendimethalin (PDM) (3,4-dimethyl-2,6-dinitro-N-pentan-3-yl-aniline) is a nitroaromatic herbicide usually applied in fields of cereals and vegetables to control the spread of broadleaf weed and annual grasses. PDM acts as a pre-emergence herbicide, which is applied to the soil before weed germination. It inhibits the step of chromosome separation during plant cell division and cell wall formation [1]. The U.S. Environmental Protection Agency classified PDM as a group C possible human carcinogen, and various studies suggest a relationship between PDM exposure and rectal, lung, and pancreatic cancers [2]. Ciprofloxacin (CIP) is a fluoroquinone antibiotic with bactericidal activity against both Gram-positive and Gram-negative bacteria [3]. CIP is used for the treatment of infectious diseases in both humans and animals. Prolonged exposure to or the excessive consumption of CIP can cause various effects including allergies, nausea, and endocrine disruption [4]. CIP is often reported to be present in the freshwater environment above the threshold [5]. Due to its complex chemical structure, CIP exhibits high thermal stability [6], contributing to its prolonged presence in the environment.

While ciprofloxacin aids in the spread of antibiotic resistance in environmental microbiota [7], pendimethalin is extremely persistent and poses dangers of bioaccumulation and phytotoxicity [8]. The presence of PDM in soil is persistent and can be somewhat remedied by the application of amendment treatment [9]. Both substances can upset the natural equilibrium and may have an adverse effect on human health by contaminating food and water. As a result, monitoring environmental exposure, evaluating possible toxicological effects, and assisting with regulatory control all depend on their sensitive and selective detection.

Potable waters are constantly monitored for the presence of any harmful substances. Expanding water monitoring would, however, give additional insight into the sources and hotspots of contamination, facilitating quicker response times and preventing the situation from becoming worse. In order to enable this widespread action, the sensing process must be portable and easy to handle. Electrochemical sensors offer these advantages, along with a low cost [10]. The materials for electrochemical sensors should meet requirements such as a high sensitivity and selectivity. The development of new materials should also fulfill the requirement of sustainability and the avoidance of secondary pollution by the material itself, when deposed after usage [11].

In this paper, a new sustainable electrode material was designed and investigated. Biochar–alumina composites, obtained by the catalytic pyrolysis of oak sawdust in the presence of alumina, were investigated as an electrode.

Biochar-based electrode materials have gained interest because they can be derived from renewable sources while still offering good electrochemical properties [12]. Biochar-based electrochemical sensors have already been used for detecting pesticides, heavy metals, and pharmaceuticals, demonstrating excellent electroanalytical performance due to their high surface area and specific functional groups [13]. On the other hand, it was shown that the presence of alumina on the electrode surface significantly influences the sensitivity of the electrode toward the analyte [14]. Further investigation showed that different electrochemical responses will be obtained depending on the alumina type [15,16].

The properties of biochar greatly depend on the parameters of the pyrolysis process. One of the approaches is catalytic pyrolysis, where the biomass is pyrolyzed in the presence of some sort of solid (clay, zeolite, metal oxides). The advantages of this process for biofuel production were investigated, revealing that it offers lower reaction temperatures, faster conversion rates, higher biofuel selectivity, and improved fuel quality [17]. The biochar–catalyst mixture obtained after pyrolysis, which has been rarely investigated [18], can have significantly changed properties, which are beneficial for application in electroanalysis.

This paper aimed to test the properties of the biochar–alumina mixture obtained after the catalytic pyrolysis of sawdust. In this study, alumina, integrated within the biochar-based composite, fulfills a dual function: it facilitates the pyrolytic process through its catalytic activity and, in synergy with biochar, enhances the electrode’s sensitivity toward the analyte. Two types of alumina were mixed with oak sawdust in three ratios and subjected to thermal treatment. The most promising composites were applied for the sensing of pesticide pendimethalin and antibiotic ciprofloxacin. The obtained results are compared with the available literature data, and the performance of the sensor presented in this paper was found to be comparable. This research demonstrates, for the first time, the use of biochar–alumina composites as electrode materials for electroanalytical applications. In contrast to traditional carbon electrodes, biochar–alumina offers an economical and environmentally friendly electrode material that uses renewable feedstocks. Selected samples were characterized by their textural, morphological, and structural properties to assess the influence of alumina type on the electroanalytical performance of the sensor.

## 2. Materials and Methods

Two types of industrial alumina from an alumina refinery (Alumina Ltd., Zvornik, Bosnia and Herzegovina) were used as the catalyst: anhydrous Al_2_O_3_ (designated as A) and trihydrate Al_2_O_3_·3H_2_O (designated as T). Oak sawdust was acquired from SME sawmill from Fruška gora, Serbia. The sawdust was dried at 120 °C for 24 h. After that, the sawdust was pyrolyzed at 400 °C for 1 h under air-limited conditions and the obtained sample was labeled as BC. The biochar–alumina composites were obtained by mixing sawdust and alumina ratios 1/1, 2/1, and 10/1 and the mixture was pyrolyzed under the same conditions. The obtained samples were designated by the type of alumina and the used ratio of sawdust and alumina: BC-A 1-1, BC-A 2-1, BC-A 10-1, BC-T 1-1, BC-T 2-1, and BC-T 10-1. Alumina samples were also calcined at the same conditions for the sake of conducting control experiments (samples designated as A and T).

The morphological, textural, and structural properties of the selected samples were assessed by SEM, N_2_ adsorption isotherm, and XRD measurement. SEM experiments were performed using the scanning electron microscope TESCAN VEGA3 (Brno, Czech Republic) operated at an accelerating voltage of 20 kV, coupled with energy-dispersive X-ray spectroscopy (EDS) (Oxford Instruments, Abingdon, UK). The samples are prepared for SEM experiments by the previous deposition of a thin film of gold onto the sample surface. The instrument Gemini 2360 Surface Area Analyzer (Micromeritics, Norcross, GA, USA) with nitrogen as the adsorptive at 77 K was used to obtain nitrogen adsorption isotherms. Before adsorption, the samples were calcined and evacuated at a temperature of 473 K in the preparation unit FlowPrep 060 (Micromeritics, Norcross, GA, USA). The isotherms were run only in the adsorption direction. The XRD analysis was performed using a Rigaku Smart Lab automatic multipurpose X-ray diffractometer (equipped with a low-background-Si sample holder support; 1D D/teX 250 Ultra detector in XRF mode) and Cu anode (*λ* = 0.1542 nm) (Rigaku, Japan). The diffractograms of the samples were obtained in the 2*θ* range from 5° to 80°, with a scanning rate of 0.3° min^−1^, and a scanning step of 0.01°.

Electrochemical experiments were conducted utilizing the Autolab electrochemical workstation (Autolab PGSTAT302N, Metrohm-Autolab BV, Barendrecht, The Netherlands) within a three-electrode cell setup. This configuration included a Ag/AgCl reference electrode in 3 M KCl, a platinum rod serving as the counter electrode, and a carbon paste electrode functioning as the working electrode. The working electrodes were prepared by hand mixing 0.1 g of carbon black (CB) (Vulcan-XC 72R) and 0.9 g of sample with 1 mL of paraffin oil. The working electrodes had the same designation as the sample used for their preparation.

The electrochemical properties were tested by recording the response of the investigated electrodes to two redox probes, 5 mM [Fe(CN)_6_]^3−/4−^ and 1 mM [Ru(NH_3_)_6_]^2+/3+^ in 0.1 M KCl, using cyclic voltammetry. The initial response of all electrodes toward pendimethalin (PDM) and ciprofloxacin (CIP) was tested in 0.1 M Britton–Robinson (BR) buffer pH 7. Selected electrodes were further tested at various pH values. The determination of PDM was performed using cyclic voltammetry (CV) and CIP using square wave voltammetry (SWV) in Britton–Robinson buffer at selected pH values. The optimized conditions used for recording curves for the calibration plot were deposition potential −1.2 V; deposition time 30 s; pulse amplitude 0.06 V; step 0.005 V; and frequency 25 Hz.

## 3. Results

### 3.1. Characterization

The characterization of selected samples was performed in order to obtain a better insight into the catalyzed pyrolysis process. Two samples, BC-A 2-1 and BC-T 2-1, were chosen in order to provide insight into the influence of the alumina type on the properties of biochar during the pyrolysis process. The characterization was performed for pure biochar (BC) and both alumina samples as a reference point.

#### 3.1.1. SEM

**Figure 1:**
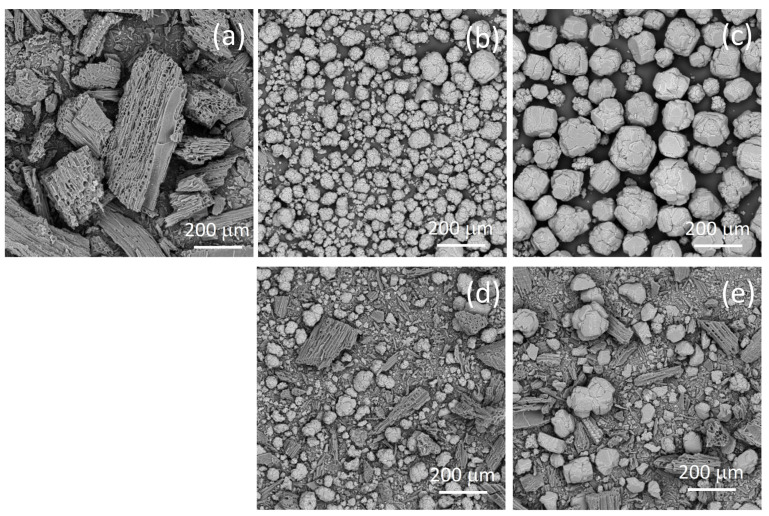
SEM micrographs of the biochar (**a**), alumina type A (**b**), alumina type T (**c**), and the biochar–alumina composites BC-A 2-1 (**d**) and BC-T 2-1 (**e**).

The morphological characteristics observed in the SEM micrographs (Figure 1) provide insight into the structural changes during pyrolysis and the interaction between biochar and different alumina types, at the same magnification (200×).

The SEM image of pure biochar BC shows a preserved wood-like structure, with aggregates of approximate sizes 100–200 μm. The porous structure of the biochar derived from lignocellulosic biomass [19] can be seen in the SEM micrograph. The SEM micrograph of alumina A shows aggregated spherical particles with rough and cracked surfaces, while the micrograph of alumina T shows large angular crystalline particles with relatively smooth but fractured surfaces.

The SEM micrographs of the composite samples show a heterogeneous morphology composed of both fibrous biochar structures and alumina particles. The composite samples show a significant amount of smaller biochar aggregates in comparison to pure biochar. In the case of the BC-T 2-1 sample, the aggregate spherical alumina particles are disrupted, as demonstrated by the smaller particle diameter compared with the pure type T alumina, while in the case of the BC-A 2-1 sample, the morphology of alumina was mostly preserved.

#### 3.1.2. N_2_ Adsorption Isotherm

**Figure 2:**
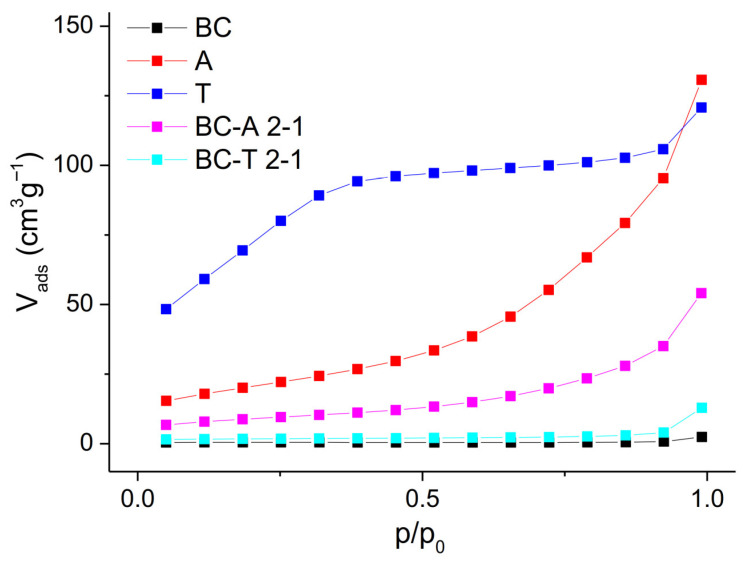
N_2_ adsorption isotherms of the biochar, alumina type A, alumina type T, and the biochar–alumina composites BC-A 2-1 and BC-T 2-1.

The nitrogen adsorption isotherms at 77 K (Figure 2) provide valuable information about the porous structure and surface area of the investigated samples.

The isotherm of pure biochar (BC) shows a nearly linear and flat profile, with minimal adsorption over the entire relative pressure range (*p*/*p*_0_). This corresponds to a Type II isotherm, which is typical of non-porous or macroporous materials [20]. Alumina A exhibits a Type IV isotherm with a significant increase in nitrogen uptake at higher relative pressures (*p*/*p*_0_ > 0.8), which is indicative of capillary condensation in mesopores. A gradual increase in adsorption across the mid-range of *p*/*p*_0_ suggests a broad distribution of mesopores. The composite BC-A 2-1 also shows a Type IV isotherm, but with a reduced adsorption volume compared with pure alumina A. The reduced uptake indicates that during pyrolysis, part of the mesoporosity of alumina is blocked or partially filled by pyrolysis-derived carbon. Alumina T displays a distinctly different isotherm, characterized by a steep initial uptake at low relative pressure (*p*/*p*_0_ < 0.1), followed by a gradual increase and eventual plateau. This behavior is indicative of combined micro- and mesoporosity and corresponds to a hybrid Type I/IV isotherm. The composite BC–T 2-1 shows a dramatic loss of porosity. The isotherm is nearly flat, with minimal nitrogen uptake, closely resembling that of pure biochar. The absence of a steep uptake suggests that the porous structure was altered during pyrolysis, specifically through the loss of micro- and mesoporosity caused by pore blocking or partial filling with carbon, as well as by the phase transformation of the trihydrate into gamma-AlOOH and eta-Al_2_O_3_. This observation is consistent with the XRD analysis results. The specific surface area and average pore diameter (calculated by the Barrett–Joyner–Halenda (BJH) method) are presented in Table 1. The dependence of cumulative pore volume on pore diameter is presented in Figure S1 in the Supplementary Materials.

A significant difference in textural properties, particularly pore size, is observed between BC-T 2-1 and the starting T-type alumina, whereas no such difference is evident for BC-A 2-1 and its corresponding A-type alumina. This suggests a stronger interaction between T-type alumina and biomass volatiles during composite formation, which may lead to the partial collapse or blockage of the porous structure, especially in the smaller pore range. This behavior can be attributed to the presence of surface –OH groups in the T-type alumina, which promote stronger interactions with the biomass, an effect not observed for A-type alumina and its composite. Consequently, the resulting surfaces are expected to exhibit different hydrophobic/hydrophilic characteristics, which in turn can influence the electrochemical performance of the materials.

#### 3.1.3. XRD

**Figure 3:**
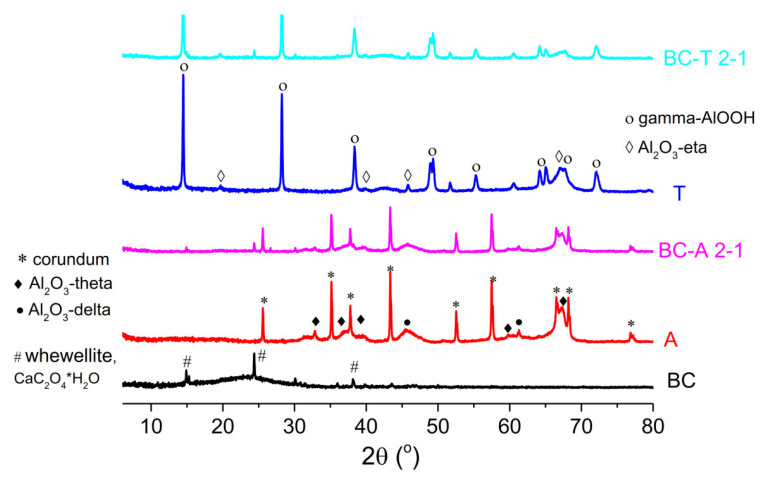
X-ray diffractograms of the biochar, alumina type A, alumina type T, and the biochar–alumina composites BC-A 2-1 and BC-T 2-1.

The XRD analysis of biochar (Figure 3) exhibited mostly amorphous characteristics with XRD reflections at 15°, 24°, and 38° corresponding to whewellite (CaC_2_O_4_•H_2_O) (JCPDS 00-020-0231). Whewellite is a mineral phase commonly found in oak trees [21].

The recorded diffraction peaks of the two alumina types, type A and type T, were sharp, indicating good crystallinity of the samples. The X-ray diffractogram of alumina A showed peaks corresponding to corundum (JCPDS 01-176-0144), Al_2_O_3_-theta (JCPDS 01-088-1609), and Al_2_O_3_-delta (JCPDS 01-079-1559), while T alumina exhibited peaks corresponding to gamma-AlOOH (JCPDS 01-072-0359) and eta-Al_2_O_3_ (JCPDS 01-079-1557). The X-ray diffractograms of the biochar–alumina composites exhibited the same peaks as the corresponding alumina type but with reduced intensity. The reduction in peak intensity was more pronounced for the composite based on T-type alumina. The decrease in peak intensity was as a consequence of the dilution of the crystalline phase by amorphous carbon and possible reduction in alumina crystallite size, as a consequence of the above-mentioned phase transformation during the pyrolysis process.

### 3.2. Electrochemical Characterization

**Figure 4:**
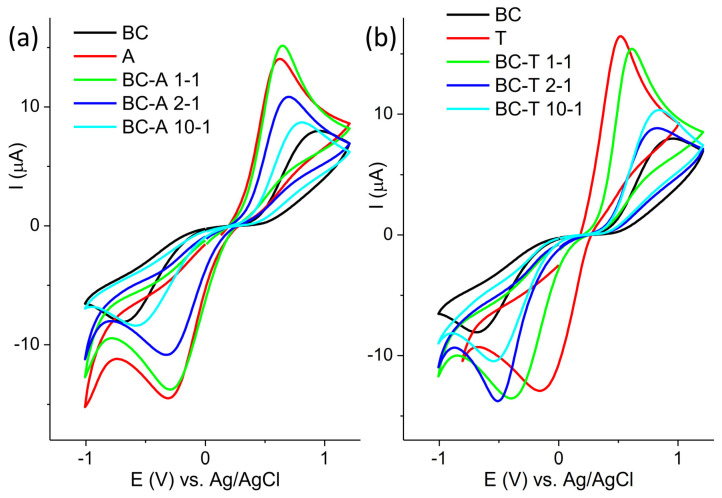
Cyclic voltammogram of (**a**) BC-A samples and (**b**) BC-T samples recorded in 5 mM [Fe(CN)_6_]^3^**^−^**^/4^**^−^** in 0.1 M KCl at scan rate of 20 mV s^−1^.

The electrochemical characterization of new electrodes is often performed by testing their response toward redox probes. The response of the investigated electrodes toward redox probe Fe[(CN)_6_]^3−/4−^ is presented in Figure 4.

The cyclic voltammograms are presented in two groups, composites obtained with the A and the T alumina, for the sake of clarity. The response of the BC electrode is presented in both groups for comparison purposes. The highest current response was obtained for the alumina samples, and the lowest for the BC sample. For both groups of samples, it can be seen that the oxidation peak potential shifted toward a more positive value and the peak current decreased with the decrease in the alumina content in the composite. The only exception was the sample BC-A 1-1, which showed a similar response to the starting alumina. The electroactive surface areas of the investigated electrodes were determined from cyclic voltammograms recorded at various scan rates using the Randles–Ševčik equation. The cyclic voltammograms and corresponding plots of peak current versus the square root of the scan rate are provided in the Supplementary Materials (Figures S2–S10). The electroactive surface areas are as follows: 0.014 cm^2^ for A; 0.19 cm^2^ for T; 0.003 cm^2^ for BC; 0.017 cm^2^ for BA-A 1-1; 0.008 cm^2^ for BC-A 2-1; 0.005 cm^2^ for BC-A 10-1; 0.015 cm^2^ for BC-T 1-1; 0.006 cm^2^ for BC-T 2-1; and 0.007 cm^2^ for BC-T 10-1.

**Figure 5:**
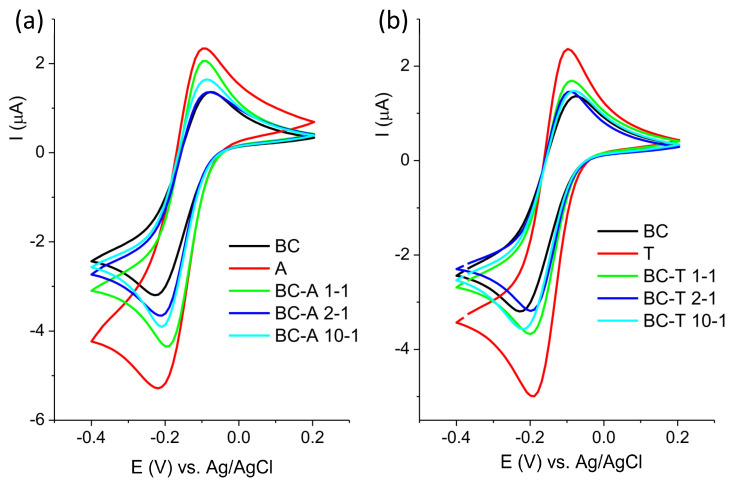
Cyclic voltammogram of (**a**) BC-A samples and (**b**) BC-T samples recorded in 1 mM [Ru(NH_3_)_6_]^2+/3+^ in 0.1 M KCl at scan rate of 20 mV s^−1^.

The response of the investigated electrodes toward redox probe Ru[(NH_3_)_6_]^2+/3+^ is presented in Figure 5. The behavior toward this probe was similar to the behavior toward Fe[(CN)_6_]^3−/−4−^, although with less pronounced differences.

The response of the electrode toward [Fe(CN)_6_]^3−/4−^ mostly depends on the composition of the electrode surface, and the response will reflect the changes in the surface chemistry [22]. On the other hand, the electron transfer of [Ru(NH_3_)_6_]^2+/3+^ proceeds without adsorption or bonding, and its response is not affected by surface functional groups, but mostly depends on the density of electronic states near the formal potential of the redox system [23].

Biochar contains various functional groups (C–H, C=O, –OH, COOH) [24]. The deprotonation of polar and acidic functionality, like –OH and –COOH, may result in a negatively charged surface that will repel the negatively charged probe. Other moieties, such as carbonyl groups, may act as electron-withdrawing sites, promoting interaction with the negatively charged probe. Alumina, on the other hand, has its own set of specific surface groups: terminal Al–OH, bridging Al–(OH)–Al, triply coordinated OH (hydroxyls bonded to three neighboring aluminum atoms), Lewis acid sites (Al^3+^), and O^2−^ surface sites [25]. The highest response toward both redox probes was obtained at the alumina-modified carbon paste electrode. In neutral or slightly acidic media (pH 5 in this experiment), protonated –OH groups and Lewis sites contribute to the formation of the positively charged surface, promoting electrostatic interaction with the negatively charged [Fe(CN)_6_]^3−/4−^ redox probe. The good response to positive [Ru(NH_3_)_6_]^2+/3+^ originated from electronic coupling, since this redox probe is less sensitive to surface charge. Surface –OH groups offer proton-coupled electron transfer (PCET) pathways, improving reversibility and kinetics [26].

### 3.3. Electroanalytical Properties

The electrocatalytic activity of the composite electrodes for detecting pendimethalin and ciprofloxacin was investigated. These substances are especially important since ciprofloxacin is a frequently given antibiotic that leads to the development of antibiotic resistance, and pendimethalin is a common herbicide that can linger in soil and water. The sensitive and selective detection of these substances is crucial because, from the perspective of xenobiotics, they are environmental contaminants that have the ability to bio-accumulate, disturb ecosystems, and have an impact on human health.

#### 3.3.1. Response of the Biochar–Alumina Composite Electrodes Toward Pendimethalin

**Figure 6:**
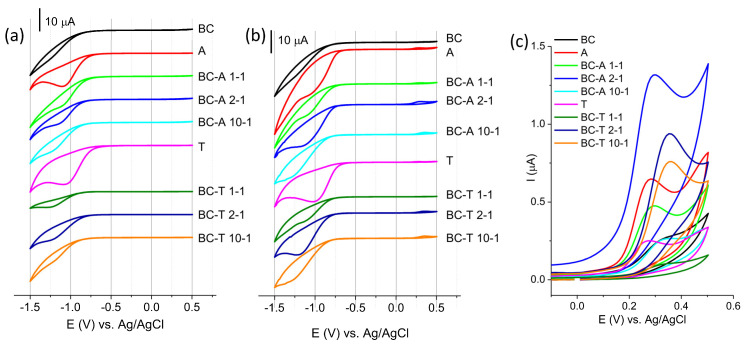
Cyclic voltammograms of investigated electrodes in (**a**) BR buffer pH 7, (**b**) BR buffer pH 7 containing 50 µM PDM, (**c**) enlarged part of CV representing oxidation of intermediate product of PDM reduction. All CV are recorded at scan rate of 50 mVs^−1^.

The response of the investigated electrodes in 0.1 M BR buffer pH 7 without and with pendimethalin is presented in Figure 6.

A characteristic feature that can be seen on all CV presented in Figure 6a is the reduction peak at potential in the region between −1.0 V and −1.2 V. The reduction peak was pronounced for the alumina sample. The alumina surface includes various species of oxides or oxy/hydroxides, Al_2_O_3_, Al(OH)_3_, and/or AlOOH. The possible origin of this peak might be the reduction of one aluminum oxy(hydroxide) species to its other form or electrodeposition of oxy(hydroxide) species. The latter reaction would be the consequence of the interfacial changes in the pH due to water electro-reduction, creating an excess of hydroxide ions [27]. The interfacial pH can reach sufficiently high values, leading to the chemical dissolution of the oxide layer, followed by oxy(hydroxide) formation on the electrode surface [28]. The surface properties of alumina are strongly influenced by the pH of the surrounding solution. Reported pK_a_ values for typical alumina phases are 6.03 for α-Al_2_O_3_, 6.78 for γ-Al(OH)_3_, and 8.50 for γ-Al_2_O_3_ [29]. In addition, various surface hydroxyl species arising from different aluminum coordination environments, such as Al_3_OH^+0.5^, Al_2_OH^+^, AlOH^+0.5^, Al_2_OH, and AlOH^−0.5^ [30], may coexist on the alumina surface, contributing to its pH-dependent behavior.

The lowest current response was recorded for BC. According to the literature, the electrochemically reducible oxygen species of carbon materials, in this potential region, are aldehyde (at 1.0 V) and epoxy (at 1.5 V) [31]. The composite biochar–alumina samples showed current responses that varied between these two margins. In addition, acidic functional groups can be present on the biochar-containing samples, including carboxylic groups (pK_a_ ≈ 3–6), lactonic groups (pK_a_ ≈ 7–9), and phenolic groups (pK_a_ ≈ 8–10) [32]. These surface functionalities contribute to the overall surface acidity and play an important role in adsorption and electrochemical interactions with target analytes.

The CV recorded in the presence of PDM (Figure 6b) showed a reduction peak in a similar potential range as that without PDM but with an increased current and additional anodic wave (the enlarged part of CV is presented in Figure 6c). The reduction peak originated from the reduction of the nitro-group of PDM, while the anodic peak originated from the oxidation of the intermediate formed during reduction [33]. The peak originating from the intermediate was selected for further investigation. The highest current response was obtained for the BC-A 2-1 electrode.

The response of the BC-A 2-1 electrode toward PDM was tested in the pH range 3–8 (Figure S1, Supplementary Material). The peak potential shifted negatively with the increase in pH. The obtained slope of 30 mVdec^−1^ indicated that the oxidation mechanism of the intermediate proceeded through a mechanism that involved exchange electrons and protons in the ratio 2:1. The highest current response was obtained at pH 7.

**Figure 7:**
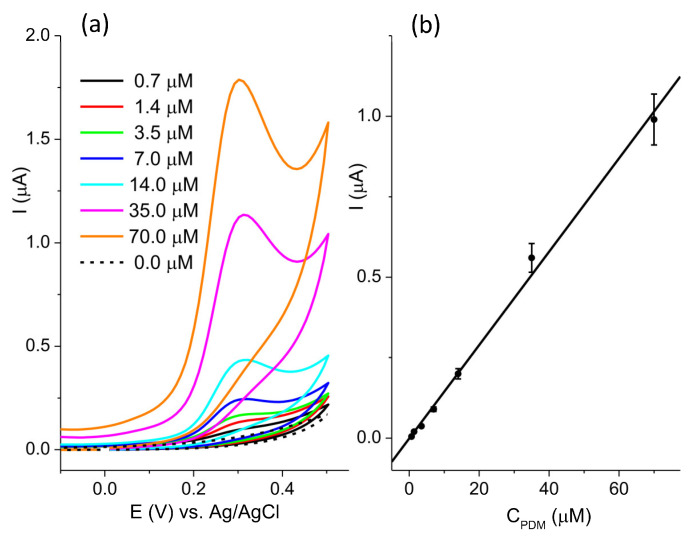
(**a**) CV recorded in the concentration range of pendimethalin from 0.7 μM to 70.0 μM in 0.1 M BR pH 7, at scan rate 50 mVs^−1^; (**b**) the calibration plot.

The sensitivity of the BC-A 2-1 electrode toward pendimethalin was investigated at pH 7 (Figure 7a). The current response was linear (Figure 7b) in the concentration range from 0.7 μM to 70.0 μM: I(μA) = −0.0009 + 0.0145 ∗ CP(μM), R^2^ = 0.995. The estimated limit of the detection, LOD, was 0.5 μM (signal-to-noise ratio = 3). The reproducibility of the BC-A 2-1 electrode was determined by measuring the current response of 20 μM PDM. The relative standard deviation (RSD) of five independently prepared electrodes was 7.9%.

The obtained results are compared with the available literature data (references [34,35,36,37] are cited in the Supplementary Materials) for PDM determination and presented in Table S1 (Supplementary Material). The data show that BC-A 2-1 exhibits a comparable or broader linear range than the best-performing sensors reported in the literature, while the LOD is adequate for practical applications. It is worth mentioning that data in the literature for electrochemical sensors for pendimethalin are not abundant.

The response of the BC-A 2-1 electrode toward PDM was tested in the presence of various interferences. The concentration of PDM was 10 µM, with the same concentration of phenol, 2-nitrophenol, and 4-nitrophenol and 25 times higher concentrations of Fe^3+^, Na^+^, Cl^−^, HCO_3_^−^, and CO_3_^2−^. The obtained results (Figure S2, Supplementary Material) showed that 4-NP had the highest interfering effect.

The commercial herbicide containing 330 g/L of pendimethalin was used as a real sample for sensor performance testing. The herbicide was diluted and an appropriate amount was added to the buffer solution. The recovery test was performed by standard addition experiments and the results are presented in Table 2.

These results indicate that the sensor provides an accurate and reliable determination of pendimethalin in real samples without significant matrix effects.

#### 3.3.2. Response the Biochar–Alumina Composite Electrodes Toward Ciprofloxacin

**Figure 8:**
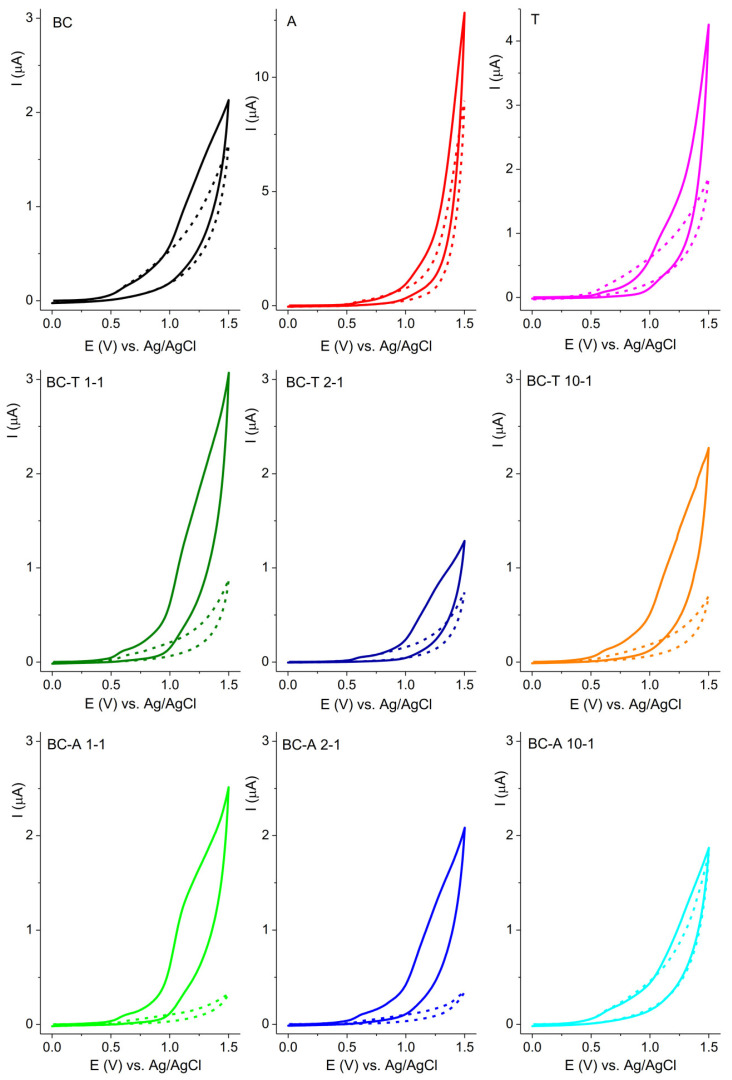
CV of investigated electrodes recorded in 0.1 M BR buffer pH 7, without (short dash curve) and with 50 μM ciprofloxacin (solid curve) at scan rate of 50 mV s^−1^.

The response of the investigated electrodes in 0.1 M BR buffer pH 7 without and with ciprofloxacin (CIP) is presented in Figure 8. The oxidation of CIP can be seen as a current rise at the foot of the oxygen evolution reaction commencing at potentials above 1.0 V vs. Ag/AgCl. The A alumina-based electrode showed the highest current response, but the T alumina-based electrode showed a better distinction of current response with regard to the background current. The current axis for all graphs presenting biochar–alumina composites was kept in the same range to enable an easier visual comparison. The best current-to-background response was obtained for the BC-A 1-1 electrode. This electrode was selected for further investigation.

The response of the BC-A 1-1 electrode toward CIP was tested in the pH range 3–8 (Figure S3, Supplementary Material). The peak potential shifted negatively with the increase in pH. The obtained slope of 59 mVs^−1^ indicated that the oxidation mechanism of CIP proceeded through a mechanism with same number of electrons and protons [38]. The highest current response was obtained at pH 5.

**Figure 9:**
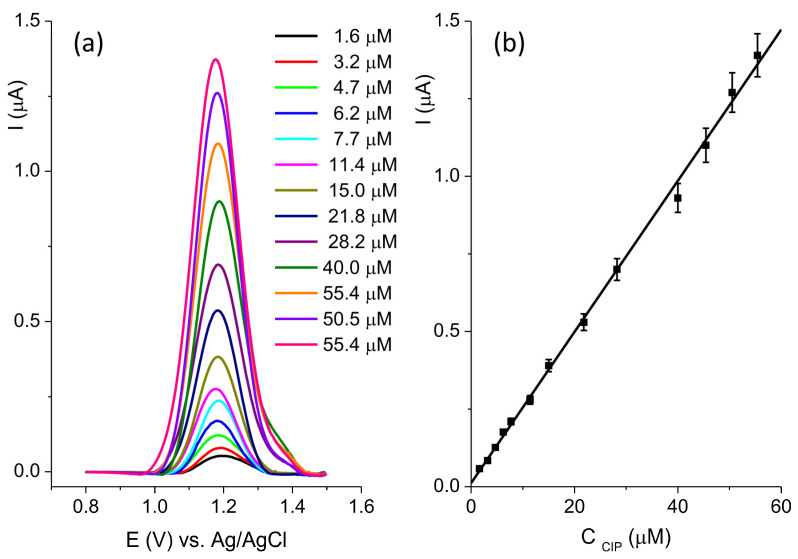
(**a**) SWV recorded at the BC-A 1-1 in the concentration range of ciprofloxacin from 1.6 μM to 55.4 μM in 0.1 M BR pH 5; (**b**) the calibration plot.

The sensitivity of the BC-A 1-1 electrode toward CIP was investigated at pH 5 using square wave voltammetry (SWV) (Figure 9a).

The optimization of the SWV method was performed and the following parameters were established: deposition potential −1.2 V; deposition time 30 s; pulse amplitude 0.06 V; step 0.005 V; and frequency 25 Hz. The current response was linear (Figure 9b) in the concentration range from 1.6 μM to 55.4 μM: I(μA) = 0.01197 + 0.02435 ∗ C_CIP_(μM), R^2^ = 0.998. The estimated limit of the detection, LOD, was 0.63 μM (signal-to-noise ratio = 3). The reproducibility of the BC-A 1-1 electrode was determined by measuring the current response of 20 μM CIP. The relative standard deviation (RSD) of five independently prepared electrodes was 6.7%.

The obtained results are compared with the available literature data (References [4,38,39,40,41,42] are cited in the Supplementary Materials) for CIP determination and presented in Table S2 (Supplementary Material). The data show that performance of the BC-A 1-1 is comparable to the literature data. It should be mentioned that the composition and preparation procedure for the biochar–alumina composite electrode is simple in comparison to some of the electrodes presented in the literature.

The response of the BC-A 1-1 electrode toward CIP was tested in the presence of various interferences. The concentration of CIP was 5 µM, with 50 times higher concentration of ascorbic acid (AA), glucose, Fe^3+^, Na^+^, Cl^−^, HCO_3_^−^, and CO_3_^2−^. The obtained results (Figure S4, Supplementary Material) showed that interfering effects were below 6%.

Commercial eye drops containing 3 mg/L of ciprofloxacin were used as a real sample for sensor performance testing. The appropriate amount of eye drops was added to the buffer solution. The recovery test was performed by standard addition experiments and the results are presented in Table 3.

The spike–recovery test demonstrates that the proposed sensor can be successfully employed for the direct analysis of real pharmaceutical samples.

The aim of this research was to investigate the possibility of producing more sustainable electrochemical sensors for two xenobiotic compounds. The comparison of electrochemical results obtained for biochar–alumina composites with different alumina types showed that the specific surface area is not a determining factor for the current response. Composites with T alumina had a significantly lower surface area than pure T alumina samples, yet the current responses for PDM and CIP were higher for composites than for alumina. The response obtained for biochar composite samples with different alumina types and the same sawdust/alumina ratio showed that the alumina type will have an influence in the determining the highest current, yet the following trend could be seen: for pendimethalin the best response was obtained for electrodes with a 2-1 ratio, while for CIP it was a 1-1 ratio. A possible explanation lies in the nature of the investigated compounds and the surface properties of the investigated composites. The detection of pendimethalin and ciprofloxacin using the biochar–alumina composite is governed by synergistic adsorption and electron transfer processes. Pendimethalin is a highly hydrophobic [43], nonpolar herbicide, so a composite with more biochar (2:1) provides a larger hydrophobic surface and better π–π interactions, favoring its detection. Ciprofloxacin, on the other hand, is more polar [44] and can form hydrogen bonds or electrostatic interactions with alumina’s hydroxyl groups and Lewis acid sites; thus, a balanced 1:1 ratio provides enough alumina to enhance these interactions without compromising conductivity too much. The combined effects of biochar’s conductivity and alumina’s catalytic properties result in enhanced electron transfer kinetics, an increased current response, and the improved sensitivity of the composite electrode toward both analytes.

It should be noted that biochar–alumina composites based on A-type alumina exhibited a better electrochemical performance toward the detection of the investigated analytes. This observation suggests that the crystalline phase present in A-type alumina, mainly corundum (i.e., α-Al_2_O_3_), is more electrochemically favorable than the γ-AlOOH phase identified in T-type alumina. The α-Al_2_O_3_ structure possesses higher crystallinity and stronger Lewis acid sites, which promote faster electron transfer and the stronger adsorption of analyte molecules at the electrode interface. In contrast, γ-AlOOH is a hydroxyl-rich, less ordered phase that tends to hinder charge transport due to surface hydroxylation and lower electronic conductivity.

## 4. Conclusions

This study shows that biochar–alumina composites derived from oak sawdust can be successfully applied to the electrochemical sensing of xenobiotics. By varying the alumina type and mixing ratio, composites with optimized surface properties were obtained, enabling the sensitive and linear detection of the two investigated xenobiotics, pendimethalin and ciprofloxacin, at low micromolar concentrations. The results presented in this research indicate the importance of surface hydrophobicity/hydrophilicity for the development of selective and sensitive electrochemical sensors. These findings indicate that biochar–alumina composites represent a promising, low-cost platform for the development of electrochemical sensors for the environmental monitoring of persistent organic pollutants.

## Figures and Tables

**Table 1:** The textural properties of samples based on the analysis of nitrogen adsorption isotherms.

Sample	S_BET_ (m^2^g^−1^)	Average Pore Diameter, BJH (nm)
BC	1.5	26.3
A	74.1	8.9
T	276.2	2.8
BC-A 2-1	31.9	9.9
BC-T 2-1	5.8	24.7

**Table 2:** Quantification of pendimethalin in real sample.

Sample	Initial Added, µM	Spiked, µM	Found, µM	Recovery, % (*n* = 3)
Commercial herbicide	5	-	4.64	92.8
	5	5	9.64	96.4
	5	10	14.45	96.3
	5	15	20.68	103.4

**Table 3:** Recovery results obtained for the analysis of ciprofloxacin in a real sample.

Sample	Initial Added, µM	Spiked, µM	Found, µM	Recovery, % (*n* = 3)
Eye drops	5	-	4.76	95.2
	5	5	9.76	97.6
	5	10	14.08	93.9
	5	15	20.54	102.7

## Data Availability

The original contributions presented in this study are included in the article/Supplementary Materials. Further inquiries can be directed to the corresponding author.

## Supplementary Materials

The following supporting information can be downloaded at: https://www.mdpi.com/article/10.3390/jox15060191/s1, Figure S1: The dependence of cumulative pore volume on the pore diameter; Figure S2. (a) Cyclic voltammograms recorded on the A electrode in 5 mM [Fe(CN)_6_]^3−/4−^ +0.1 M KCl at the scan rate of 10 mVs^−1^, 20 mVs^−1^, 50 mVs^−1^, 100 mVs^−1^, 150 mVs^−1^ and 200 mVs^−1^. The arrow labels the increase of the scan rate. (b) The dependence of the anodic peak current on the square root of scan rate. Figure S3. (a) Cyclic voltammograms recorded on the T electrode in 5 mM [Fe(CN)_6_]^3−/4−^ +0.1 M KCl at the scan rate of 10 mVs^−1^, 20 mVs^−1^, 50 mVs^−1^, 100 mVs^−1^, 150 mVs^−1^ and 200 mVs^−1^. The arrow labels the increase of the scan rate. (b) The dependence of the anodic peak current on the square root of scan rate. Figure S4. (a) Cyclic voltammograms recorded on the BC electrode in 5 mM [Fe(CN)_6_]^3−/4−^ +0.1 M KCl at the scan rate of 10 mVs^−1^, 20 mVs^−1^, 50 mVs^−1^, 100 mVs^−1^, 150 mVs^−1^ and 200 mVs^−1^. The arrow labels the increase of the scan rate. (b) The dependence of the anodic peak current on the square root of scan rate. Figure S5. (a) Cyclic voltammograms recorded on the BC-A 1-1 electrode in 5 mM [Fe(CN)_6_]^3−/4−^ +0.1 M KCl at the scan rate of 10 mVs^−1^, 20 mVs^−1^, 50 mVs^−1^, 100 mVs^−1^, 150 mVs^−1^ and 200 mVs^−1^. The arrow labels the increase of the scan rate. (b) The dependence of the anodic peak current on the square root of scan rate. Figure S6. (a) Cyclic voltammograms recorded on the BC-A 2-1 electrode in 5 mM [Fe(CN)_6_]^3−/4−^ +0.1 M KCl at the scan rate of 10 mVs^−1^, 20 mVs^−1^, 50 mVs^−1^, 100 mVs^−1^, 150 mVs^−1^ and 200 mVs^−1^. The arrow labels the increase of the scan rate. (b) The dependence of the anodic peak current on the square root of scan rate. Figure S7. (a) Cyclic voltammograms recorded on the BC-A 10-1 electrode in 5 mM [Fe(CN)_6_]^3−/4−^ +0.1 M KCl at the scan rate of 10 mVs^−1^, 20 mVs^−1^, 50 mVs^−1^, 100 mVs^−1^, 150 mVs^−1^ and 200 mVs^−1^. The arrow labels the increase of the scan rate. (b) The dependence of the anodic peak current on the square root of scan rate. Figure S8. (a) Cyclic voltammograms recorded on the BC-T 1-1 electrode in 5 mM [Fe(CN)_6_]^3−/4−^ +0.1 M KCl at the scan rate of 10 mVs^−1^, 20 mVs^−1^, 50 mVs^−1^, 100 mVs^−1^, 150 mVs^−1^ and 200 mVs^−1^. The arrow labels the increase of the scan rate. (b) The dependence of the anodic peak current on the square root of scan rate. Figure S9. (a) Cyclic voltammograms recorded on the BC-T 2-1 electrode in 5 mM [Fe(CN)_6_]^3−/4−^ +0.1 M KCl at the scan rate of 10 mVs^−1^, 20 mVs^−1^, 50 mVs^−1^, 100 mVs^−1^, 150 mVs^−1^ and 200 mVs^−1^. The arrow labels the increase of the scan rate. (b) The dependence of the anodic peak current on the square root of scan rate. Figure S10. (a) Cyclic voltammograms recorded on the BC-T 10-1 electrode in 5 mM [Fe(CN)_6_]^3−/4−^ +0.1 M KCl at the scan rate of 10 mVs^−1^, 20 mVs^−1^, 50 mVs^−1^, 100 mVs^−1^, 150 mVs^−1^ and 200 mVs^−1^. The arrow labels the increase of the scan rate. (b) The dependence of the anodic peak current on the square root of scan rate. Figure S11: (a) Cyclic voltammograms recorded on BC-A 2-1 electrode in BR buffer at diffrent pH in range 3-8 with concentration of PDM; (b) Dependence of current (I) and potential (E) on pH for the oxidation peak of intermediere. The values of I are shown on the left axis, while those of E are shown on the right axis. Table S1: Comparison of electrochemical sensors for determination of pendimethalin; Figure S12: The response of the BC-A 2-1 electrode to pendimethalin in the presence of interfering species. Concetration of pendimethalin was 10 µM, with the same concentration of phenol, 2-nitrophenol and 4-nitrophenol and concentration of 250 µM of Fe^3+^, Na^+^, Cl^−^, HCO_3_^−^ and CO_3_^2−^. Figure S13: (a) Cyclic voltammograms recorded on BC-A 1-1 electrode in BR buffer at diffrent pH in range 3-7; (b) plot of dependence of I and E on pH. Table S2: Comparison of electrochemical sensors for determination of ciprofloxacin. Figure S14: The response of the BC-A 1-1 electrode to ciprofloxacion in the presence of interfering species. Concentration of ciprofloxacine was 5 µM, and concentration of ascorbic acid (AA), glucose, Fe^3+^, Na^+^, Cl^−^, HCO_3_^−^ and CO_3_^2−^ was 250 µM.

## Abbreviations

The following abbreviations are used in this manuscript:

SEM	Scanning electron microscopy
XRD	X-ray diffraction
LOD	Limit of detection
PDM	Pendimethalin
CIP	Ciprofloxacin
CV	Cyclic voltammetry
BR buffer	Britton–Robinson buffer
SWV	Square wave voltammetry
BJH	Barrett–Joyner–Halenda method
