# Supplementary material for: Sustainable Biochar–Alumina Composites for Electroanalytical Sensing of Herbicide and Antibiotic
Source: J Xenobiot. 2025 Nov 10;15(6):191. doi: 10.3390/jox15060191 (PMC12641625; doi:10.3390/jox15060191)
Supplement: Supplementary file 1 [file jox-15-00191-s001.zip › jox-3956768-supplementary.pdf]

Journal of *Xenobiotics*

MDPI

Supplementary Materials: Sustainable Biochar–Alumina Composites for Electroanalytical Sensing of Herbicide and Antibiotic

Nataša Jović-Jovičić, Tatjana Novaković, Tanja Barudžija, Marija Ajduković, Natalia Czerwinska, Chiara Giosuè and Zorica Mojović

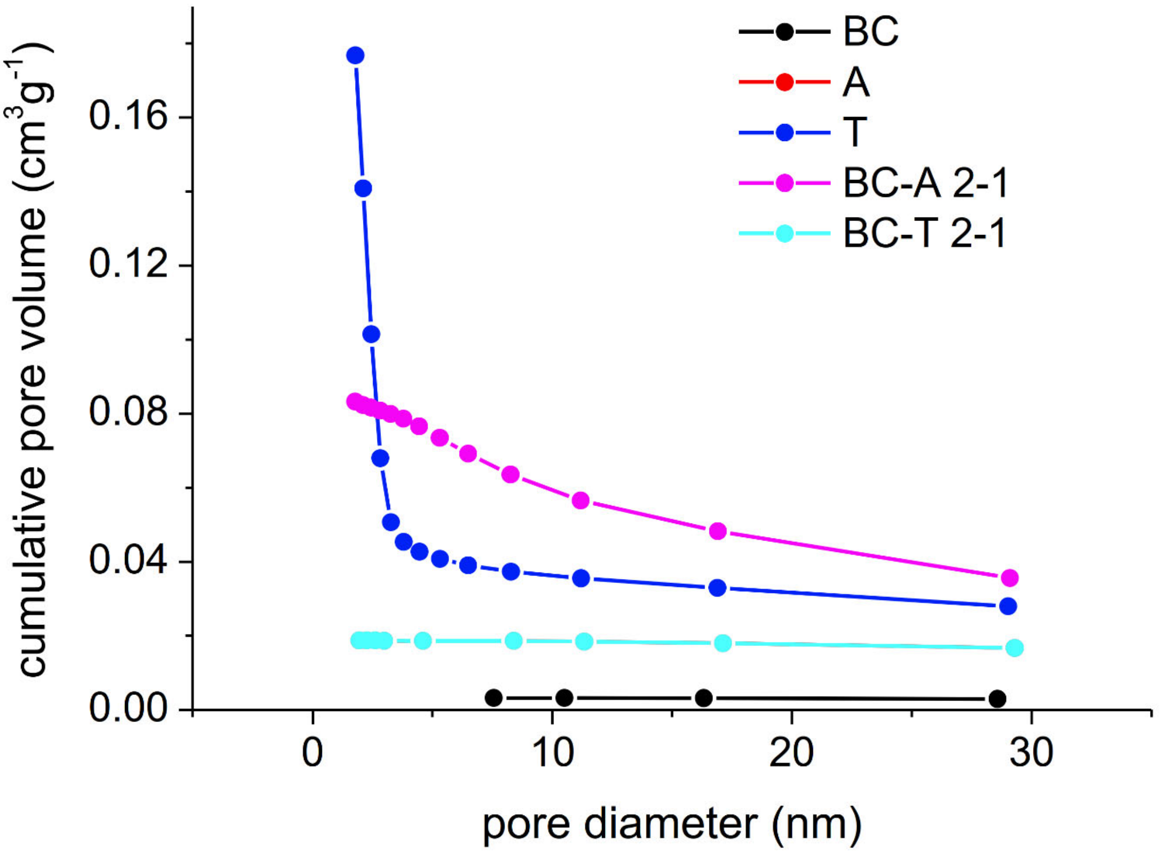

Figure S1: The dependence of cumulative pore volume on the pore diameter

BJH analysis was applied and cumulative pore volume and surface area were used to calculate average pore diameter using equation:

$d_p = 4V_p/A$, where d_p is average pore diameter, V_p is cumulative pore volume in m^3/g and A is cumulative pore area in m^2/g.

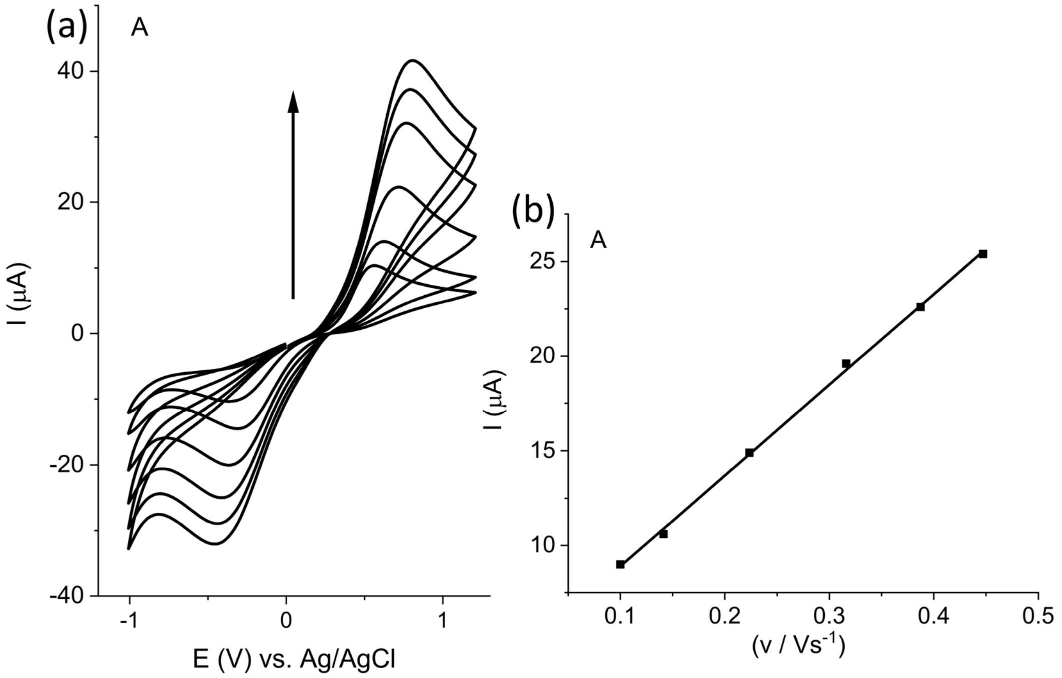

Figure S2. a) Cyclic voltammograms recorded on the A electrode in 5 mM $[Fe(CN)_6]^{3-/4-}$ +0.1 M KCl at the scan rate of 10 mVs^{-1}, 20 mVs^{-1}, 50 mVs^{-1}, 100 mVs^{-1}, 150 mVs^{-1} and 200 mVs^{-1}. The arrow labels the increase of the scan rate. b) The dependence of the anodic peak current on the square root of scan rate.

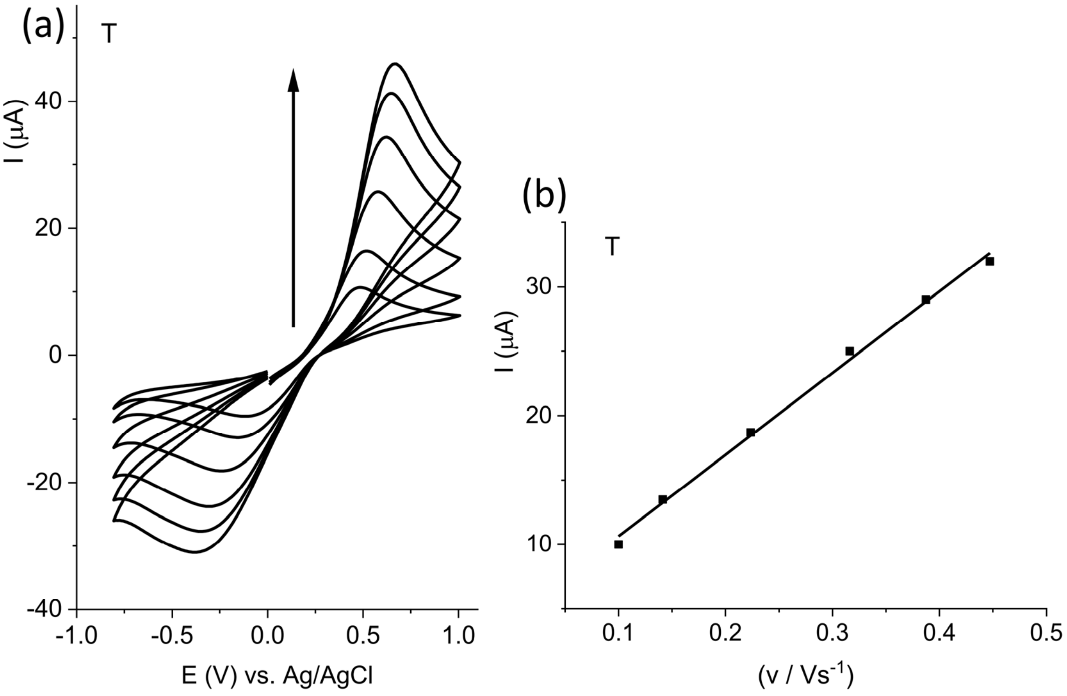

Figure S3. a) Cyclic voltammograms recorded on the T electrode in 5 mM $[Fe(CN)_6]^{3-/4-}$ +0.1 M KCl at the scan rate of 10 mVs^{-1}, 20 mVs^{-1}, 50 mVs^{-1}, 100 mVs^{-1}, 150 mVs^{-1} and 200 mVs^{-1}. The arrow labels the increase of the scan rate.b) The dependence of the anodic peak current on the square root of scan rate.

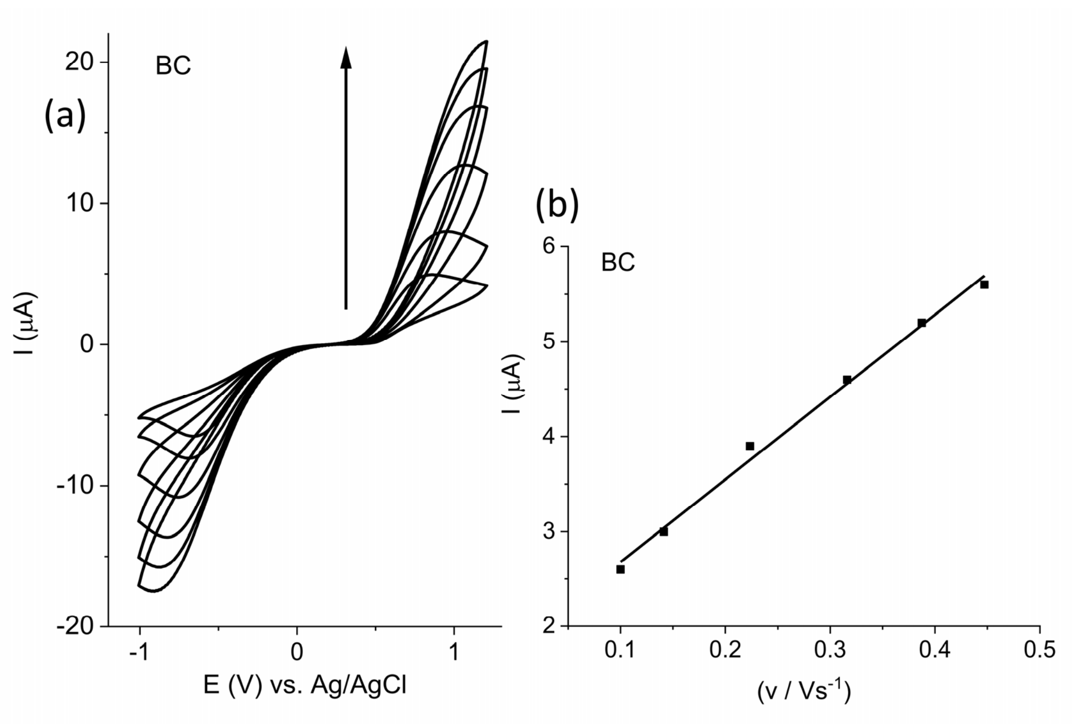

Figure S4. a) Cyclic voltammograms recorded on the BC electrode in 5 mM $[Fe(CN)_6]^{3-/4-}$ +0.1 M KCl at the scan rate of 10 mVs^{-1}, 20 mVs^{-1}, 50 mVs^{-1}, 100 mVs^{-1}, 150 mVs^{-1} and 200 mVs^{-1}. The arrow labels the increase of the scan rate. b) The dependence of the anodic peak current on the square root of scan rate.

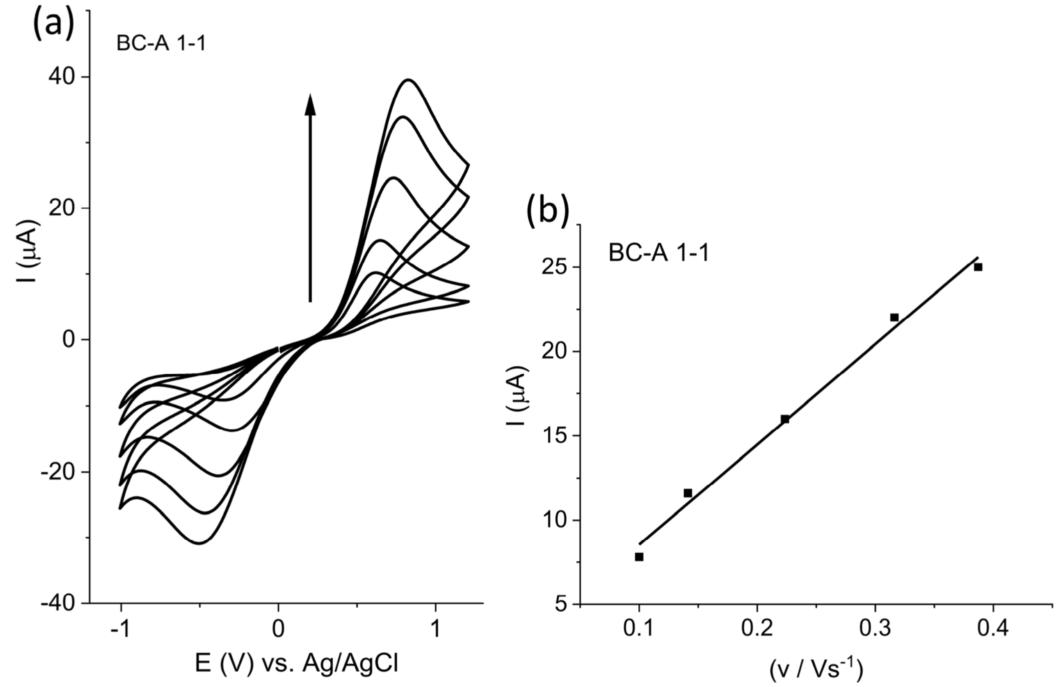

Figure S5. a) Cyclic voltammograms recorded on the BC-A 1-1 electrode in 5 mM $[Fe(CN)_6]^{3-/4-}$ +0.1 M KCl at the scan rate of 10 mVs^{-1}, 20 mVs^{-1}, 50 mVs^{-1}, 100 mVs^{-1}, 150 mVs^{-1} and 200 mVs^{-1}. The arrow labels the increase of the scan rate. b) The dependence of the anodic peak current on the square root of scan rate.

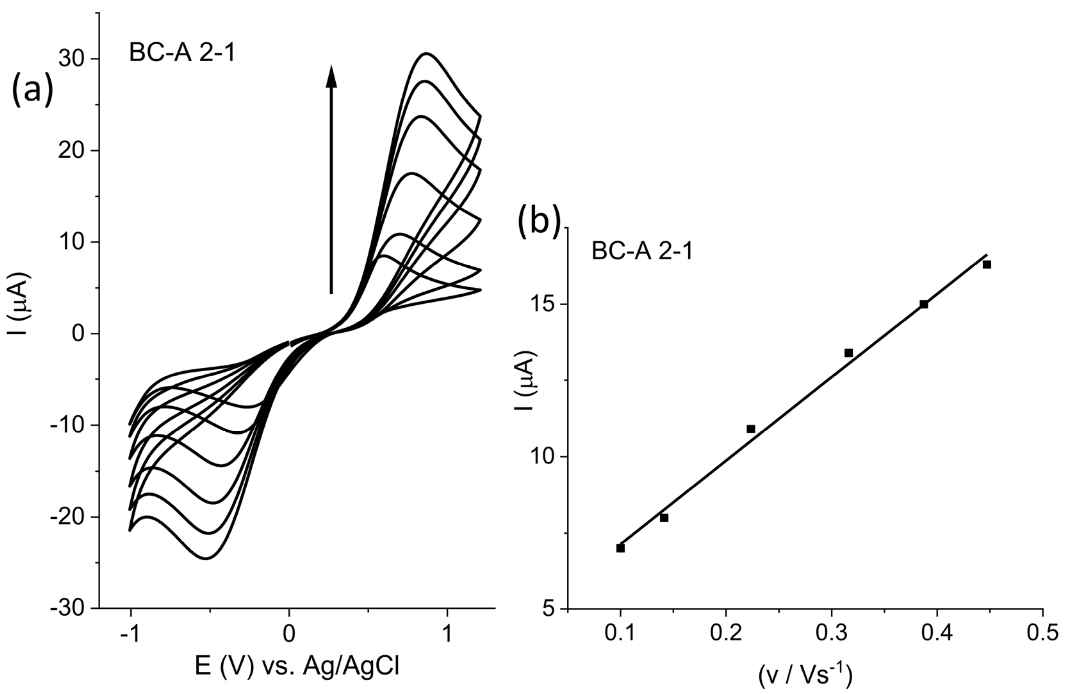

Figure S6. a) Cyclic voltammograms recorded on the BC-A 2-1 electrode in 5 mM $[Fe(CN)_6]^{3-/4-}$ +0.1 M KCl at the scan rate of 10 mVs^{-1}, 20 mVs^{-1}, 50 mVs^{-1}, 100 mVs^{-1}, 150 mVs^{-1} and 200 mVs^{-1}. The arrow labels the increase of the scan rate. b) The dependence of the anodic peak current on the square root of scan rate.

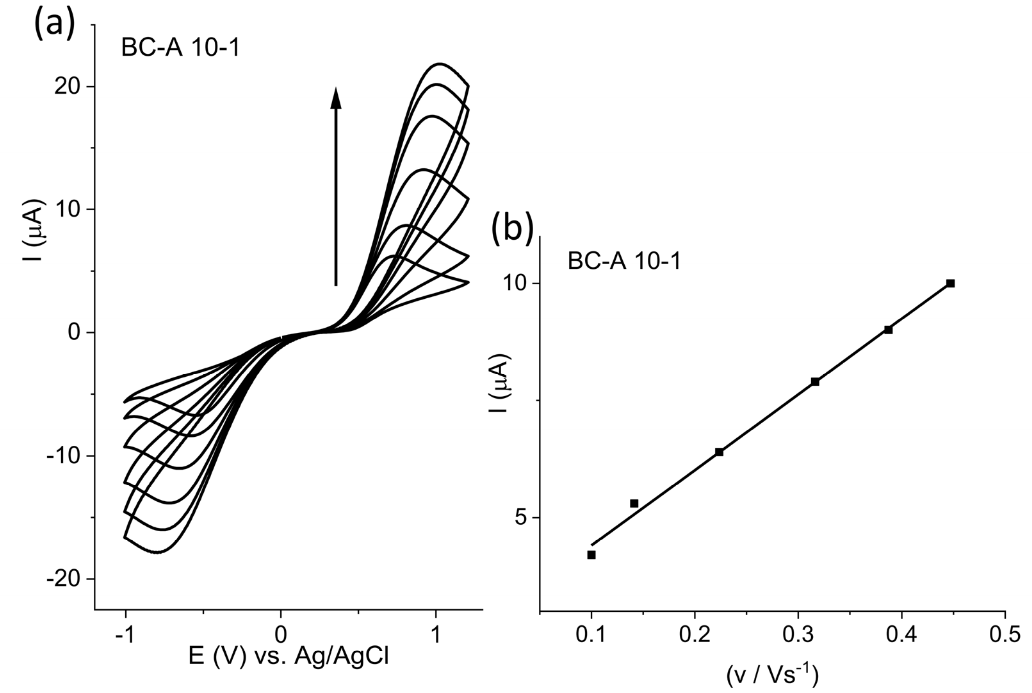

Figure S7. a) Cyclic voltammograms recorded on the BC-A 10-1 electrode in 5 mM $[Fe(CN)_6]^{3-/4-}$ +0.1 M KCl at the scan rate of 10 mVs^{-1}, 20 mVs^{-1}, 50 mVs^{-1}, 100 mVs^{-1}, 150 mVs^{-1} and 200 mVs^{-1}. The arrow labels the increase of the scan rate. b) The dependence of the anodic peak current on the square root of scan rate.

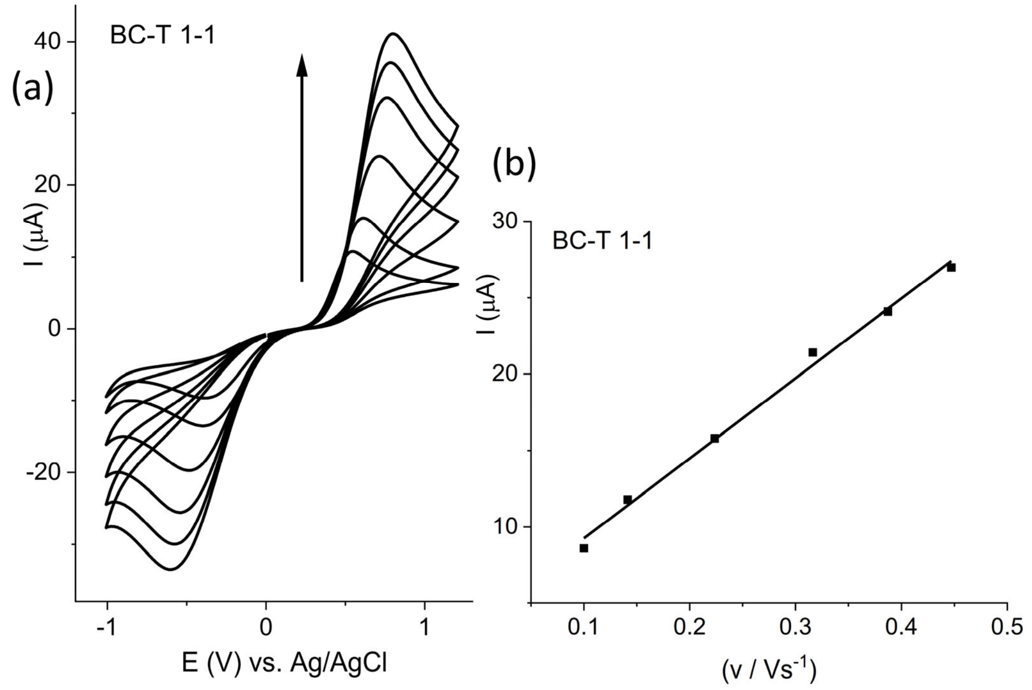

Figure S8. a) Cyclic voltammograms recorded on the BC-T 1-1 electrode in 5 mM $[Fe(CN)_6]^{3-/4-}$ +0.1 M KCl at the scan rate of 10 mVs^{-1}, 20 mVs^{-1}, 50 mVs^{-1}, 100 mVs^{-1}, 150 mVs^{-1} and 200 mVs^{-1}. The arrow labels the increase of the scan rate. b) The dependence of the anodic peak current on the square root of scan rate.

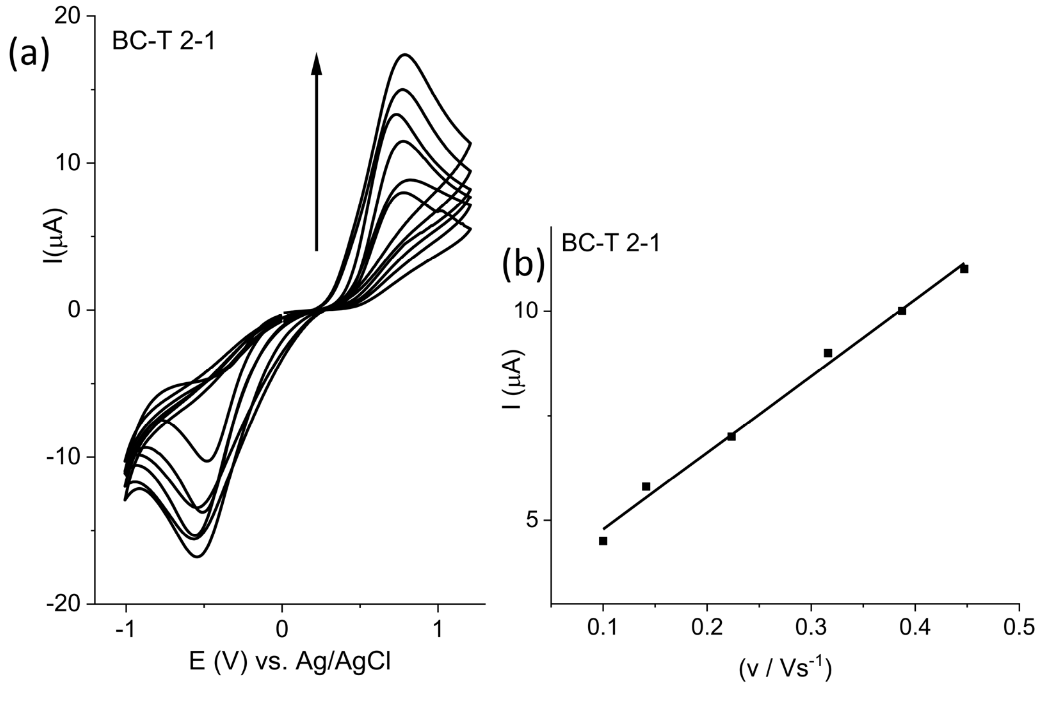

Figure S9. a) Cyclic voltammograms recorded on the BC-T 2-1 electrode in 5 mM $[Fe(CN)_6]^{3-/4-}$ +0.1 M KCl at the scan rate of 10 mVs^{-1}, 20 mVs^{-1}, 50 mVs^{-1}, 100 mVs^{-1}, 150 mVs^{-1} and 200 mVs^{-1}. The arrow labels the increase of the scan rate. b) The dependence of the anodic peak current on the square root of scan rate.

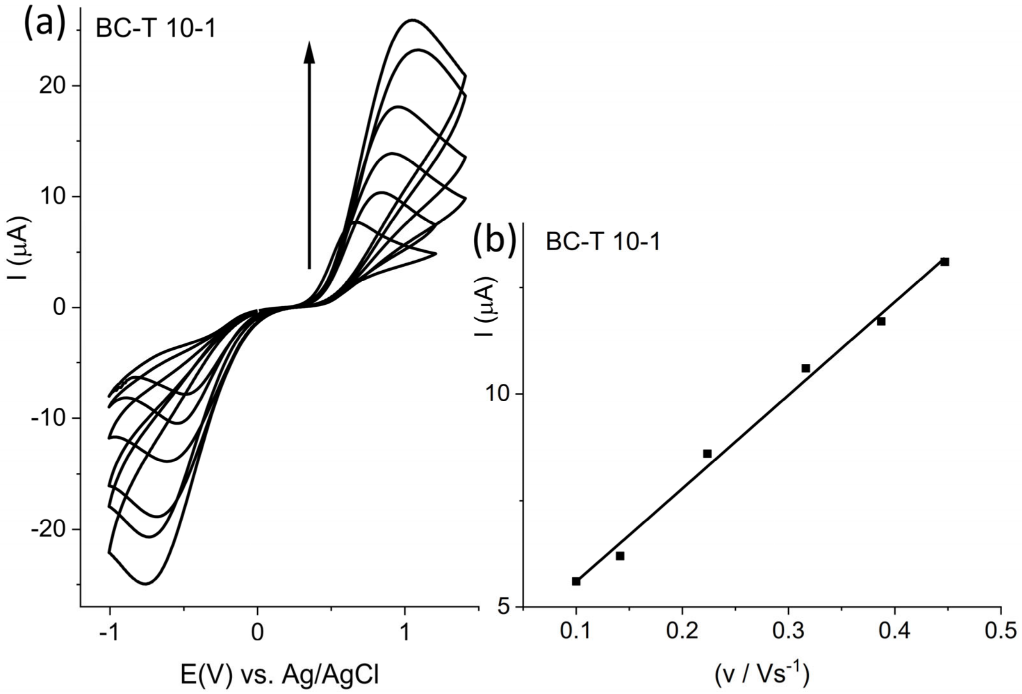

Figure S10. a) Cyclic voltammograms recorded on the BC-T 10-1 electrode in 5 mM $[Fe(CN)_6]^{3-/4-}$ +0.1 M KCl at the scan rate of 10 mVs^{-1}, 20 mVs^{-1}, 50 mVs^{-1}, 100 mVs^{-1}, 150 mVs^{-1} and 200 mVs^{-1}. The arrow labels the increase of the scan rate. b) The dependence of the anodic peak current on the square root of scan rate.

The response of the BC-A 2-1 electrode toward PDM was tested in pH range 3-8 (Figure S2) The peak potential shifted negatively with the increase of pH and the slope of 30 $mVdec^{-1}$ indicates that the oxidation mechanism of intermediate proceeded through mechanism that involved exchange electrons and protons in the ratio 2:1. The highest current response was obtained for the pH 7.

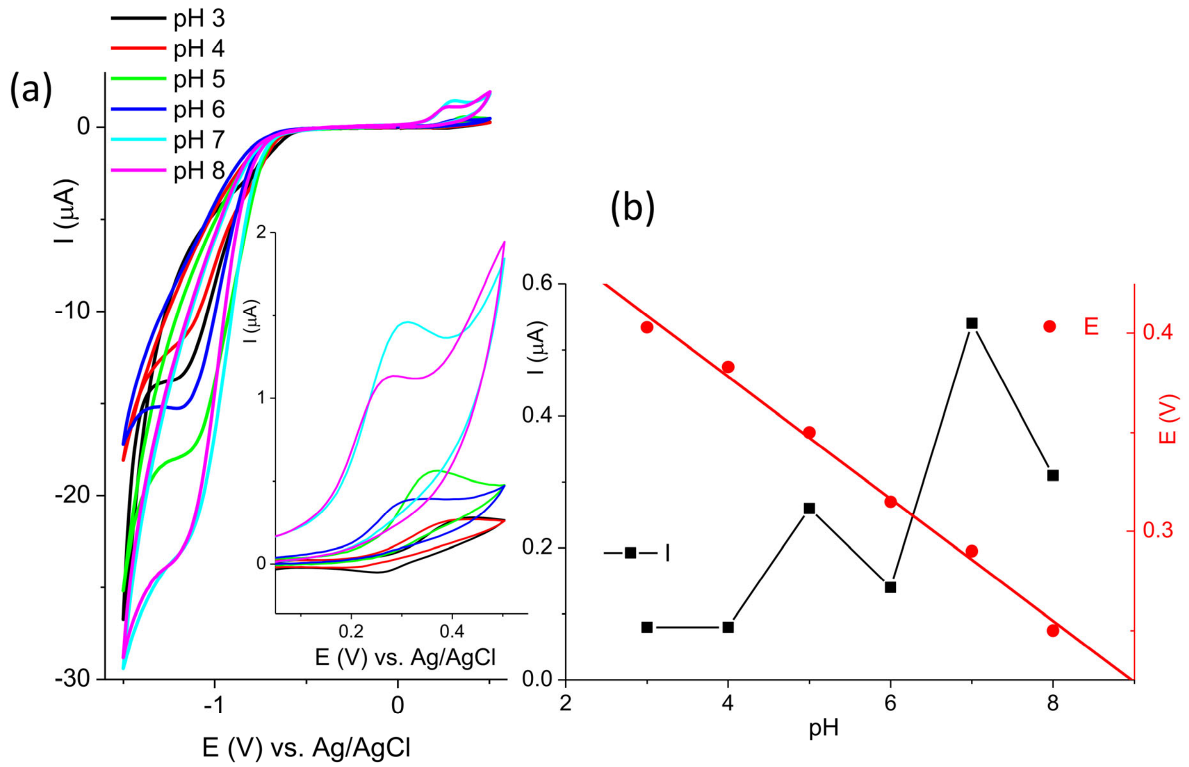

Figure S11: a) Cyclic voltammograms recorded on BC-A 2-1 electrode in BR buffer at diffrent pH in range 3-8 with concentration of PDM; b) Dependence of current (I) and potential (E) on pH for the oxidation peak of intermediere. The values of I are shown on the left axis, while those of E are shown on the right axis.

Table S1: Comparison of electrochemical sensors for determination of pendimethalin

Electrode	Linear range (μM)	LOD (μM)	reference
Silver nanoparticle-modified electrode	0.07 - 2	0.036	[1]
ZIF-8/Co/ rGO/C_3N_4/ds-DNA/SPCE	0.01–35	0.008	[2]
BiFE	0.3 – 1.0	0.037	[3]
Nafion-Graphene modified GCE	1.76 – 401	8.69	[4]
BC-A 2-1	0.7 – 70.0	0.5	This work

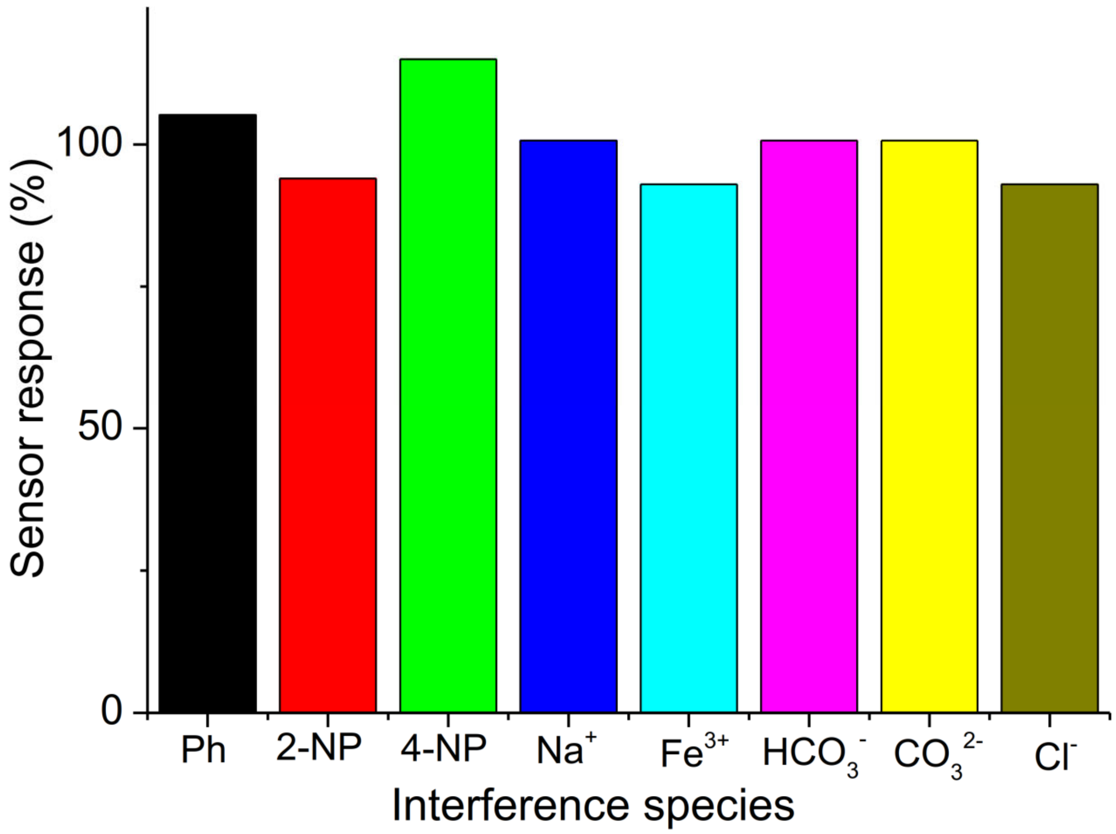

Figure S12: The response of the BC-A 2-1 electrode to pendimethalin in the presence of interfering species. Concetration of pendimethalin was 10 μM, with the same concentration of phenol, 2-nitrophenol and 4-nitrophenol and concentration of 250 μM of Fe^{3+}, Na^{+}, Cl^{-}, HCO_3^{-} and CO_3^{2-}.

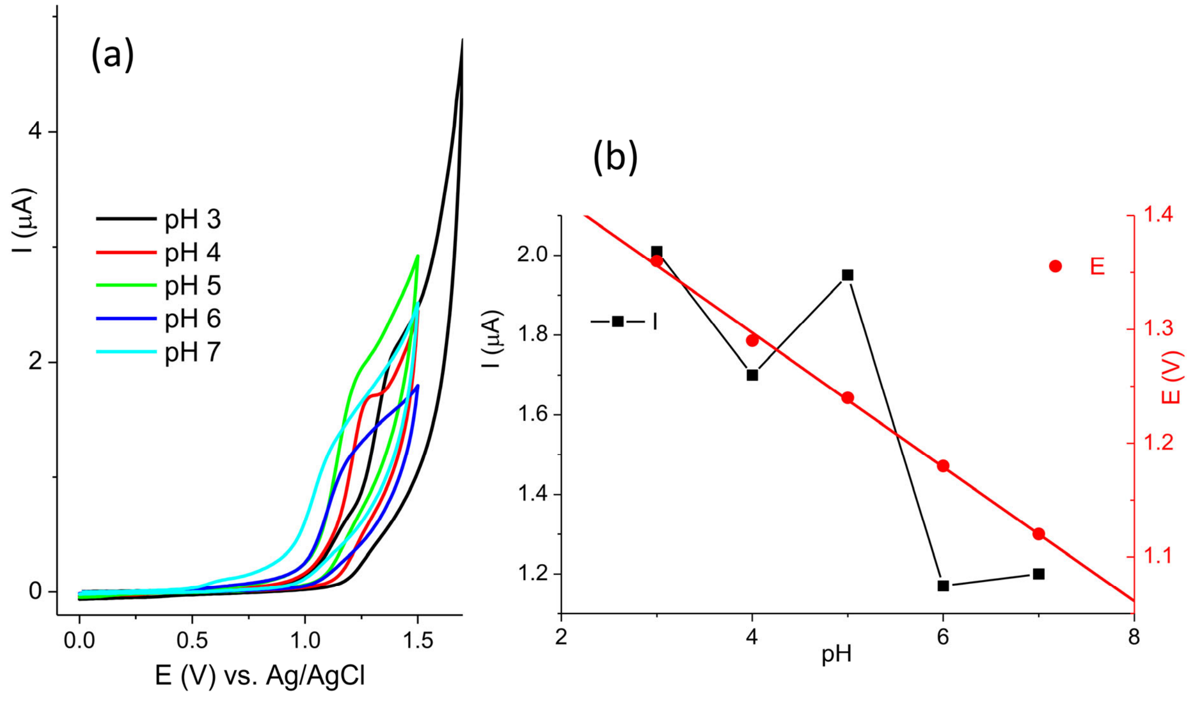

Figure S13: a) Cyclic voltammograms recorded on BC-A 1-1 electrode in BR buffer at diffrent pH in range 3-7; b) plot of dependence of I and E on pH

Table 2: Comparison of electrochemical sensors for determination of ciprofloxacin

Electrode	Linear range (μM)	LOD (μM)	reference
AgNPs-CB-Ch/GCE	3.1-24.8<br>36.9-130.3	0.48	[5]
3D CB–PLA	1.0 – 12.5	0.3	[6]
N–F/CPE	1.0–18	1.0	[7]
CIMMO/rGO/GCE	0.00075–0.10	0.00047	[8]
ERGO/PANI/PARS/SPCE	0.01 to 69.8	0.0021	[9]
SUPRAS-AuNPs-AC/GCE	0.0005–0.025	0.0002	[10]
BC-A 1-1	1.6 - 55.4	0.63	This work

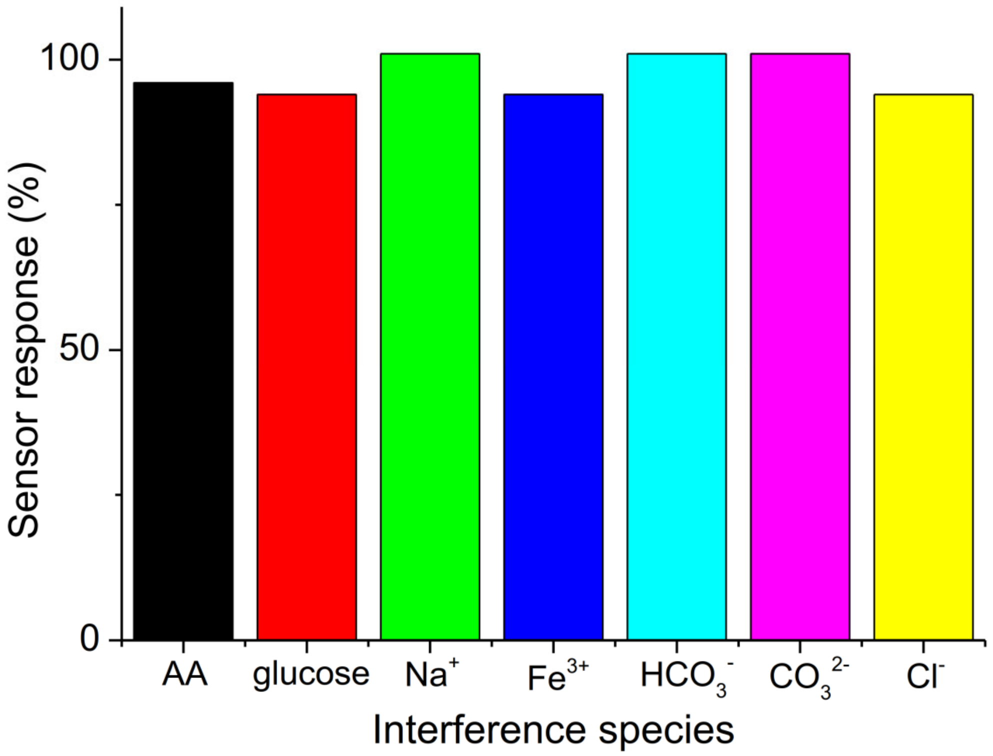

Figure S14: The response of the BC-A 1-1 electrode to ciprofloxacion in the presence of interfering species. Concentration of ciprofloxacine was 5 μM, and concentration of ascorbic acid (AA), glucose, Fe^{3+}, Na^{+}, Cl^{-}, HCO_3^{-} and CO_3^{2-} was 250 μM.

[1] de Lima, C. A.; Santana, E. R.; Piovesan, J. V.; Spinelli, A. Silver nanoparticle-modified electrode for the determination of nitro compound-containing pesticides, *Anal. Bioanal. Chem*. **2016,** *408*, 2595-2606. https://doi.org/10.1007/s00216-016-9367-5
[2] Karimi-Maleha, H.; Liub, Y.; Lib, Z.; Darabia, R.; Oroojic, Y.; Karaman, C.; Karimi, F.; Baghayeri, M.; Rouhi, J.; Fu, L.; Rostamnia, S.; Rajendran, S.; Sanati, A. L.; Sadeghifar, H.; Ghalkhani, M. Calf thymus ds-DNA intercalation with pendimethalin herbicide at the surface of ZIF-8/Co/rGO/C3N4/ds-DNA/SPCE; A bio-sensing approach for pendimethalin quantification confirmed by molecular docking study. *Chemosphere* **2023**, *332*, 138815. https://doi.org/10.1016/j.chemosphere.2023.138815
[3] Gerent, G. G.; Gonçalves, C. Q.; da Silva, P. S.; Spinelli, A. In situ bismuth-film electrode for square-wave cathodic voltammetric detection of pendimethalin at nanomolar level. *Electrochim. Acta* **2015**, *168*, 379–385. https://doi.org/10.1016/j.electacta.2015.03.207
[4] Koçak, B.; Çelikkan, H. Voltammetric Determination of Pendimethalin with Nafion-Graphene Modified Glassy Carbon Electrode. *Karaelmas Science and Engineering Journal* **2021**, *11*, 98-107. https://doi.org/10.7212/karaelmasfen.825084
[5] Meireles, L. M.; Silva R. M.; da Silva, R. C.; Okumura, L. L.; Moreira, R. P. L.; Silva, T. A. Low-cost electrochemical sensor for ciprofloxacin antibiotic based on green-synthesized silver nanoparticles and carbon black. *J. Solid State Electrochem.* **2025**, *29*, 3111–3122. https://doi.org/10.1007/s10008-024-06033-y

[6] Henriques, B. F.; Neumann, A.; Bertolim, L. V.; de Freitas, R. C.; Silva, L. R. G.; Stefano, J. S.; Janegitz, B. C. Development and Application of 3D-Printed Electrochemical Sensors for Ciprofloxacin Detection. *Electroanalysis* **2025**, *37*, e12008. https://doi.org/10.1002/elan.12008

[7] Hernandez, P.; Aguilar-Lira, G. Y.; Islas, G.; Rodriguez, J. A. Development of a new voltammetric methodology for the deter mination of ciprofloxacin in beef samples using a carbon paste electrode modified with nafion and fullerenes. *Electroanalysis* **2021**, *33*, 1539–1546. https://doi.org/10.1002/elan.202060525

[8] Chuiprasert, J.; Srinives, S.; Boontanon, N.; Polprasert, C.; Ramungul, N.; Karawek, A.; Boontanon, S. K. Ciprofloxacin Electrochemical Sensor Using Copper–Iron Mixed Metal Oxides Nanoparticles/Reduced Graphene Oxide Composite. *ACS Omega* **2024**, *9*, 23172–23183. https://doi.org/10.1021/acsomega.3c06705

[9] Elancheziyan, M.; Lee, S.; Yoon, T. H.; Singh, M.; Lee, D.; Won, K. Disposable electrochemical sensors based on reduced graphene oxide/polyaniline/poly(alizarin red S)-modified integrated carbon electrodes for the detection of ciprofloxacin in milk. *Microchim Acta* **2024**, *191*, 507. https://doi.org/10.1007/s00604-024-06578-9

[10] Gissawong, N.; Srijaranai, S.; Boonchiangma, S.; Uppachai, P.; Seehamart, K.; Jantrasee, S.; Moore, E.; Mukdasai, S. An elec trochemical sensor for voltammetric detection of ciprofloxacin using a glassy carbon electrode modified with activated carbon, gold nanoparticles and supramolecular solvent. *Microchim Acta* **2021**, *188*, 208. https://doi.org/10.1007/s00604-021-04869-z
